# Supplementary material for: Splice-Junction-Based Mapping of Alternative Isoforms in the Human Proteome
Source: Cell Rep. Author manuscript; Available in PMC 2020 Jan 15. (PMC6961840; doi:10.1016/j.celrep.2019.11.026)
Supplement: 2 [file NIHMS1546469-supplement-2.zip › DF1/PXD006675/LeftAtrium/LeftAtrium_33_BBS1_EGQSAPLLSAHVNMPGSEGLAAPNRPLNPE.pdf]

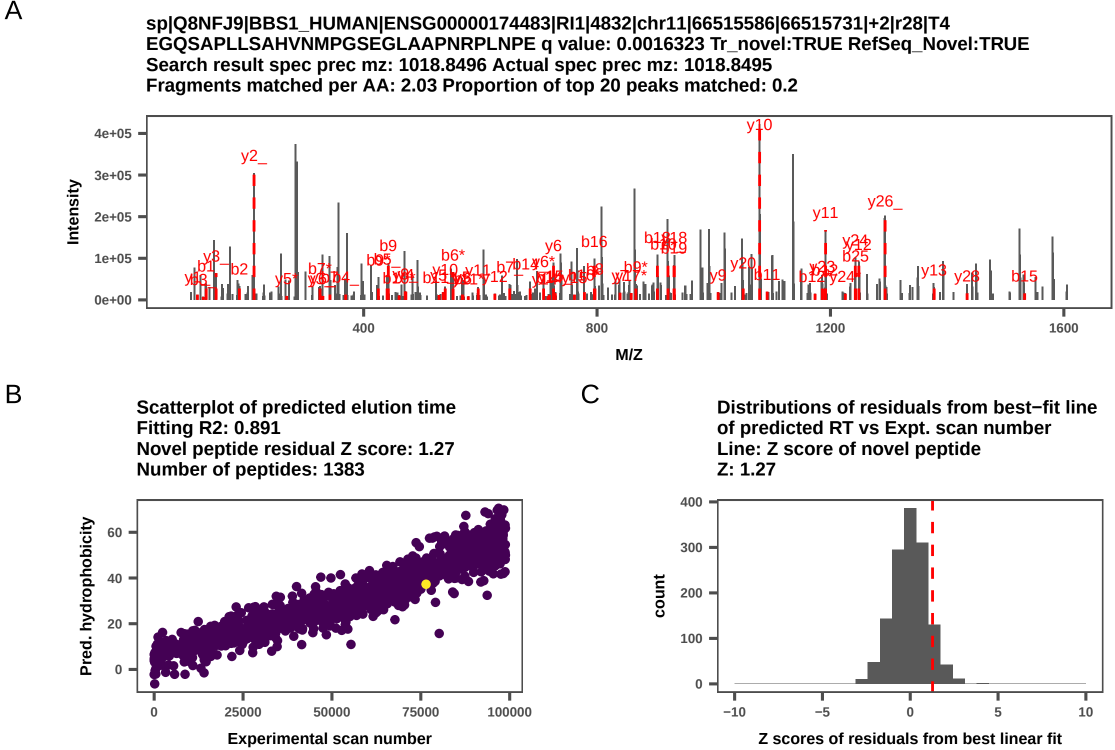

A
sp|Q8NFJ9|BBS1_HUMAN|ENSG00000174483|RI1|4832|chr11|66515586|66515731|+2|r28|T4
EGQSAPLLSAHVNMPGSEGLAAPNRPLNPE q value: 0.0016323 Tr_novel:TRUE RefSeq_Novel:TRUE
Search result spec prec mz: 1018.8496 Actual spec prec mz: 1018.8495
Fragments matched per AA: 2.03 Proportion of top 20 peaks matched: 0.2
Intensity
4e+05
3e+05
2e+05
1e+05
0e+00
y10
y2_
y11
y26_
b16
y6
b9
b6*
y13
y28
b15
y9
b11
b25
y20
M/Z
400
800
1200
1600
B
Scatterplot of predicted elution time
Fitting R2: 0.891
Novel peptide residual Z score: 1.27
Number of peptides: 1383
Pred. hydrophobicity
60
40
20
0
0
25000
50000
75000
100000
Experimental scan number
C
Distributions of residuals from best−fit line
of predicted RT vs Expt. scan number
Line: Z score of novel peptide
Z: 1.27
count
400
300
200
100
0
−10
−5
0
5
10
Z scores of residuals from best linear fit
